# Kaempferol Protects Blood Vessels From Damage Induced by Oxidative Stress and Inflammation in Association With the Nrf2/HO-1 Signaling Pathway

**DOI:** 10.3389/fphar.2020.01118

**Published:** 2020-07-22

**Authors:** He Yao, Jingyu Sun, Jie Wei, Xin Zhang, Bing Chen, Yajun Lin

**Affiliations:** ^1^ The Key Laboratory of Geriatrics, Beijing Hospital, National Center of Gerontology, Beijing, China; ^2^ Institute of Basic Theory for Chinese Medicine, China Academy of Chinese Medical Sciences, Beijing, China; ^3^ The Key Laboratory of Geriatrics, Peking University Fifth School of Clinical Medicine, Beijing, China

**Keywords:** inflammation, kaempferol, Nrf2, oxidative stress, vascular injury

## Abstract

Over recent years, an increasing number of studies have confirmed that the occurrence and development of vascular pathological changes are closely related to oxidative stress and the inflammatory response of the vascular endothelium. Kaempferol is the most common flavonoid compound found in fruits and vegetables. Our present research identified that kaempferol had the capability to protect the vascular endothelium in a mouse model of vascular injury and explored the specific mechanisms underlying these effects by investigating oxidative stress, the extent of cardiovascular injury, and inflammatory markers such as NF-κB, TNF-α, IL-6, and the Nrf2/HO-1 signaling pathway. Analysis showed that kaempferol reduced oxidative stress and inflammation mediated by H_2_O_2_ and paraquat, respectively, both *in vitro* and *in vivo*. Furthermore, kaempferol suppressed the levels of TNF-α and IL-6, and the activation of NF-κB, in aortic tissues and human umbilical vein endothelial cells (HUVECs). Finally, we observed that kaempferol corrected the levels of antioxidants and elevated the protein levels of Nrf2 and HO-1 in aortic tissues and HUVECs. Collectively, our findings prove that kaempferol protects blood vessels from damage induced by oxidative stress and inflammation and that the Nrf2/HO-1 signaling pathway plays a key role in mediating these effects.

## Introduction

Vascular injury plays an important role in many cardiovascular diseases (Bruder-Nascimento et al., 2019). The dysfunction and activation of endothelial cells induced by various factors leads to the production of pro-inflammatory cytokines and reactive oxygen species; these factors are both important causes of vascular injury (Catapano et al., 2017). A large body of experimental and clinical evidence now indicates that vascular inflammation plays an important role in the development of endothelial dysfunction. Interactions between the pro-inflammatory and pro-oxidative environments in the vascular system can increase the risk of cardiovascular disease as such interactions work in concert to accelerate the formation of atherosclerotic plaques (Hajjar and Gotto, 2013; Assar et al., 2016).

Flavonoids are a class of compounds widely found in plants. Among the different subclasses of flavonols, the most common compounds are quercetin, myricetin, and kaempferol (Filomeni et al., 2010). Of these, kaempferol (Kae) is well known for its pharmacological ability to reduce oxidative stress and inflammation in various organs and tissues (Kampkotter et al., 2007; Park et al., 2009; Ren et al., 2019). Recent studies have demonstrated the protective effects of Kae on the heart by regulating nuclear factor erythroid-2 related factor (Nrf2) and nuclear factor kappa B (NF-κB) (Suchal et al., 2016; Chen et al., 2018; Zhang et al., 2019). Of these factors, Nrf2, a master transcription factor, is transferred to the nucleus after activation, where it binds to the upstream cis-regulated antioxidant response element (ARE) sequence located in the promoter regions of genes responsible for cell protection (David et al., 2017). Nrf2 subsequently activates a variety of cellular protective enzymes, such as catalase (CAT), superoxide dismutase (SOD), glutathione (GSH-Px), and heme oxygenase-1 (HO-1) (Abdou et al., 2019). There is mounting evidence that the Nrf2 signaling pathway may represent a potential target for fighting oxidative stress-related cardiovascular diseases due to its significant antioxidant properties.

NF-κB is present in the cytoplasm along with IκBα, an inhibitory protein. Previous research has demonstrated that NF-κB is activated by the phosphorylation of IκBα, thereby triggering the expression of genes associated with inflammation (Wang et al., 2020). The activation of NF-κB subsequently induces the expression of proinflammatory cytokines, including nitric oxide synthase (iNOS), COX-2, tumor necrosis factor (TNF-α), interleukin 1 beta (IL-1β), and interleukin-6 (IL-6) (Rojas et al., 2013; Liang et al., 2018). Recent studies have further confirmed that Kae inhibits the activation of NF-κB (Kadioglu et al., 2015). However, the effect of Kae on the oxidative-stress-induced activation of NF-κB in vascular endothelial cells remains unknown.

In brief, although Kae can effectively relieve oxidative damage and inflammation in a variety of organs, only a small number of studies have confirmed its effect on vascular damage. Recent studies have shown that Kae can reduce doxorubicin-induced endothelial toxicity injury, and that this protective effect is related to the inhibition of oxidative stress (Wu et al., 2020). However, it is unclear whether Kae can protect against vascular damage induced by other pro-oxidative factors. To address this outstanding question, we attempted to investigate the anti-inflammatory and anti-oxidant effects of Kae and explore the specific mechanisms underlying such effects. Identification of the specific anti-inflammatory and antioxidant effect of Kae should allow its development as a candidate compound to prevent and treat oxidative damage to blood vessels.

## Materials and Methods

### Reagents and Chemicals

Paraquat, thiazolyl blue tetrazolium bromide (MTT), and dimethyl sulfoxide (DMSO), were purchased from Sigma-Aldrich (Darmstadt, Germany) and kaempferol (98% purity) was purchased from Aladdin Biochemical Technology Co., Ltd. (Shanghai, China). All of the kits used to measure indicators of oxidative stress (including malondialdehyde [MDA], SOD, GSH-Px, total antioxidant capacity [T-AOC] and CAT) were purchased from Nanjing Jiancheng Institute of Biological Engineering (Nanjing, China). We also purchased a DCFH-DA fluorescent probe and an SA-β-gal staining kit from Beyotime Biotechnology (Shanghai, China).

### Preparation of Kae Solution and Suspension

Kae solution was used in all *in vitro* experiments. First, we prepared a stock solution of Kae in DMSO. This stock solution was then diluted in culture medium to the required concentration, and DMSO was added to ensure that the concentration of DMSO in each group was consistent at 0.1%. For the control group, 0.1% DMSO was also added to the culture medium. For *in vivo* experiments, we prepared a suspension of Kae with DMSO and normal saline; the final concentration of DMSO was maintained at 1% in each group. The suspension was shaken well prior to administration to experimental mice *via* the intragastric route. The control group was also given 1% DMSO dissolved in normal saline.

### Cell Culture

Human umbilical vein cells (HUVECs) were obtained from ScienCell Research Laboratories Inc. (US) and grown in endothelial cell medium (ECM) containing 5% fetal bovine serum (FBS) and 1% penicillin-streptomycin in 5% CO_2_ at 37°C.

### Cell Viability Assays

Cell viability was evaluated by MTT assays. HUVECs were seeded in 96-well plates (3 × 10^3^ cells/well). The cells were then treated with Kae (2.5, 5, and 10 μmol/l) for 24 h or treated with H_2_O_2_ (100 μmol/l) plus Kae (2.5, 5, and 10 μmol/l) for 24 h. After 24 h, MTT solution (5 mg/ml; 20 μmol/l) was added and then incubated for 4 h. Finally, the supernatants were removed and 150 µl of DMSO was added to dissolve the formazan crystals. The optical density (OD) was then measured at a wavelength of 570 nm using a microplate reader (Multiskan™ MK3; Thermo Fisher Scientific, Inc., US), and cell viability was calculated.

### Determination of Reactive Oxygen Species (ROS)

Intracellular ROS production was assessed by measuring the fluorescence intensity of 2,7-dichlorodi-hydrofluorescein diacetate (DCFH-DA). HUVECs were seeded in 24-well plates (1 × 10^4^ cells/well). Then, the cells were treated with 100 μmol/l H_2_O_2_ or treated with H_2_O_2_ (100 μmol/l) plus 5 μmol/l Kae for 24 h. After 24 h, the cells were washed three times in phosphate-buffered saline (PBS) and then loaded with a DCFH-DA probe (1:1,000, 10 min) at 37°C in a CO_2_ incubator. After rinsing in PBS, images were finally acquired by fluorescence microscopy (Olympus, Tokyo, Japan).

### Senescence-Associated-β-Galactosidase (SA-β-Gal) Staining Assays

HUVECs were treated with Kae (2.5 and 5 μmol/l) for 24 h or treated with H_2_O_2_ (100 μmol/l) plus Kae (2.5 and 5 μmol/l) for 24 h. After 24 h, the cell culture medium was removed. Then, the cells were washed once with PBS and fixed at room temperature for 15 min. Following the removal of cell fixative, the cells were washed with PBS three times (for 3 min each time). Next, we added 1 ml of working solution (10 μl of dyeing solution A + 10 μl dyeing solution B + 930 μl dyeing solution C + 50 μl of X-Gal solution) to the plate and incubated for 24 h at 37˚C in 5% CO_2_. Cell staining was observed by light microscopy. The proportion (%) of positive-stained cells out of the total number of cells was then determined in three random fields by light microscopy at 10× and 20× magnification.

### Animals, Groups, and Dosing

*In vivo* experiments were performed with 8-week-old male C57 BL/6J mice, weighing approximately 23 g. The animals are provided by Sipeifu Biotechnology (Beijing, China). The study was approved by the Animal Ethics Committee of Beijing Hospital. The animals were maintained at a temperature of 22 ± 3°C and a relative humidity of 50 ± 10% with a 12 h dark/light cycle. Experimental animals were provided with standard food and water. The animals were divided into five groups (six mice in each group), as follows: Group 1 (vehicle-treated, control) received 1% DMSO by daily gavage for 10 consecutive days and an intraperitoneal injection of normal saline every 3 days; Group 2 (Kae-treated) received Kae (25 mg/kg body weight) by daily gavage for 10 consecutive days and an intraperitoneal injection of normal saline every 3 days; Group 3 (paraquat-treated) received 1% DMSO by daily gavage for 10 consecutive days and an intraperitoneal injection of paraquat (10 mg/kg body weight) every 3 days; Group 4 (paraquat + Kae-25) received Kae (25 mg/kg body weight) by daily gavage for 10 consecutive days and an intraperitoneal injection of paraquat (10 mg/kg body weight) every 3 days; Group 5 (paraquat + Kae-50) received Kae (50 mg/kg body weight) by daily gavage for 10 consecutive days and an intraperitoneal injection of paraquat (10 mg/kg body weight) every 3 days. At the end of treatment, we recorded body weight, heart weight, and blood was collected from the abdominal aorta according to standard procedures. Blood was centrifuged (3,000 rpm for 15 min) and the serum was stored at −4°C to await analysis. After the mice were sacrificed, the abdominal aorta was removed and cryopreserved at −80°C to await further analysis.

### Evaluation of Oxidative Stress

Adherent HUVECs in each group were collected with a scraper and dissolved in 150 μl of PBS. The collected cells were then broken by ultrasound on ice. Then, we determined the levels of various antioxidant indicators, including MDA, SOD, GSH-Px, CAT, and T-AOC, using the above kits in accordance with the manufacturer’s guidelines. The concentration of MDA and the relative activities of SOD and GSH-Px in the serum were also analyzed, as described above.

### Preparation of Total Protein From Abdominal Aorta Tissues

Abdominal aorta tissues were homogenized in a tissue homogenizer using PBS and the homogenates were centrifuged at 12,000 rpm for 10 min to obtain the supernatants. Next, we used RIPA lysis buffer to extract total protein in accordance with the manufacturer’s guidelines. Total protein content was then determined by a protein assay kit (Sigma-Aldrich, US).

### Immunoblot Analysis

Total protein extracts were then used in immunoblotting experiments with a variety of antibodies, including GAPDH (1:1,000, CST, US), TNF-α (1:1,000, CST, US), IL-6 (1:1,000, CST, US), NF-κB p65 (1:1,000, CST, US), Nrf2 (1:1,000, Proteintech, US), HO-1(1:1,000, Proteintech, US), and KNDC1 (1:1,000, Sigma-Aldrich, US). The proteins were analyzed using enhanced chemiluminescence western blotting detection reagents (GE Healthcare, US). ImageJ software version 1.0 was then used to quantify the optical density of each protein band normalized to the that of the internal control (GAPDH).

### Statistical Analysis

All statistical analysis was conducted using GraphPad Prism 5.0. Data are presented as mean ± standard deviation (SD). One-way analysis of variance (ANOVA) with Tukey’s *post-hoc* test was used to determine the statistical significance of multiple comparisons. P values < 0.05 were considered to represent statistically significant results.

## Results

### The Cytoprotective Effect of Kae on the Senescence of HUVECs Induced by H_2_O_2_

**Figure 1:**
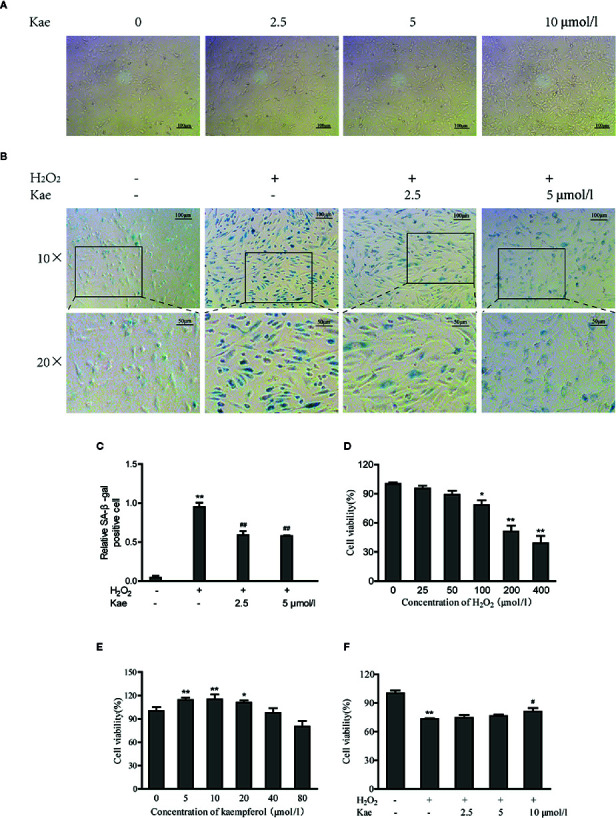
The protective effects of Kae on human umbilical vein endothelial cells (HUVECs). **(A)** Images captured from HUVECs grown in the presence of 0, 2.5, 5, and 10 μmol/l Kae for 24 h (Magnification: 10×). **(B, C)** HUVECs were incubated with 0, 2.5, and 5 μmol/l Kae and 100 μmol/l H_2_O_2_ for 24 h. Relative levels of senescence were then detected by SA-β-gal staining under light microscopy (Magnification: 10×, 20×). HUVECs were then treated with various concentrations of H_2_O_2_ **(D)** or Kae **(E)** or both Kae (0, 2.5, 5, and 10 μmol/l) and 100 μmol/l H_2_O_2_ **(F)** at 37°C for 24 h; viability was then determined by MTT assays (three biological replicates). *P < 0.05 and **P < 0.01, compared with the control group. ^#^P < 0.05 and ^##^P < 0.01, compared with the H_2_O_2_-treated group.

Observation of the growth of HUVECs in the presence of Kae showed that when the concentration of Kae was 5 μmol/l, the number of cells increased significantly. However, when the concentration of Kae reached 10 μmol/l, the morphology of the cells changed significantly (**Figure 1A**). When HUVECs were treated with 100 μmol/l of H_2_O_2_ for 24 h, the number of cells that were positive for SA-β-gal staining increased significantly. Observation of cell morphology showed that the cells had undergone significant changes. For example, cells had become spherical, cell volume had increased, the number of connections between cells had decreased, cell growth was slow, and the cell density had decreased. However, after the addition of 2.5 and 5 μmol/l of Kae, along with 100 μmol/l of H_2_O_2_, we observed that the number of positive cells decreased, the morphology and arrangement of cells were clearly regular, the growth status was significantly improved, and the number of cells was increased (**Figures 1B, C**). Data showed that the application of H_2_O_2_ at a concentration of 100 μmol/l or above could significantly reduce the viability of HUVECs compared with that of the control group (**Figure 1D**). However, compared with the group treated with 100 μmol/l of H_2_O_2,_ the cell viability of the group treated with 10 μmol/l of Kae, and the group treated with 100 μmol/l of H_2_O_2_, was significantly increased (**Figure 1F**). In addition, concentrations of Kae below10 μmol/l also increased the viability of normal HUVECs. However, when the concentration exceeded 20 μmol/l, this effect began to diminish (**Figure 1E**).

### Kae Reduced the Levels of ROS and Increased the Levels of Antioxidant Indicators *In Vitro*

**Figure 2:**
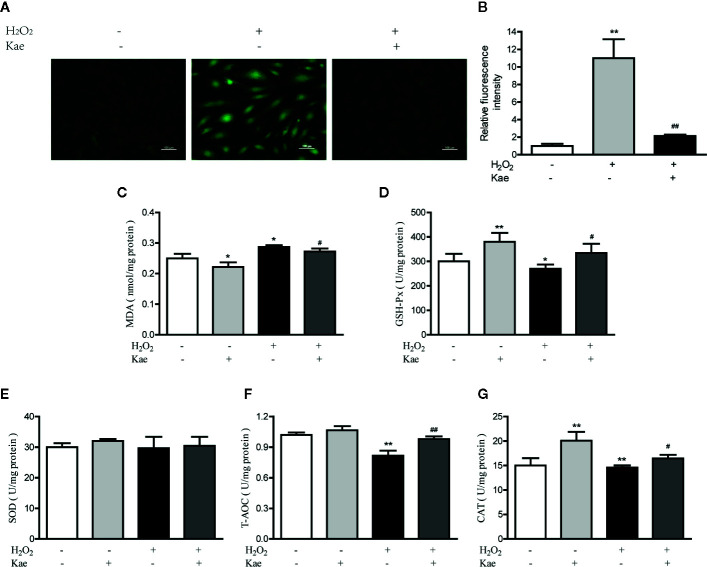
Kae reduced ROS and increased the levels of oxidant indicators *in vitro*. **(A–B)** HUVECs were treated with or without 100 μmol/l H_2_O_2_ for 24 h with 24 h exposure to 5 μmol/l Kae. The intracellular levels of ROS were then measured by fluorescence microscopy (10×). The concentration of MDA **(C)** and the activities of GSH-Px **(D)**, SOD **(E)**, T-AOC **(F)**, and CAT (G), were then determined using appropriate kits after treating HUVECs for 24 h with 100 µmol/l and 5 μmol/l of H_2_O_2_ and Kae, respectively. Data are presented as the mean ± standard deviation (SD) of three independent experiments. *P < 0.05 and **P < 0.01, compared with the control group. ^#^P < 0.05 and ^##^P < 0.01, compared with the H_2_O_2_-treated group.

Oxidative stress can lead to the excessive production of ROS, thus leading to an imbalance between the oxidation and antioxidant systems. We measured ROS levels in HUVECs after the addition of H_2_O_2_ with or without Kae (**Figures 2A, B**). Analysis showed that the addition of H_2_O_2_ increased the intracellular levels of ROS levels by 10-fold when compared with that in the control group; treatment with 5 μmol/l Kae caused a significant reduction in the upregulated ROS levels induced by H_2_O_2_. By investigating markers of oxidative stress, we found that H_2_O_2_ treatment significantly inhibited the antioxidant defense mechanism of the HUVECs. It was also evident that the concentration of MDA increased significantly, whereas the levels or activities of T-AOC and GSH-Px were significantly reduced compared with those of the control group. By treating HUVECs with 5 μmol/l Kae, we were able to significantly reverse this effect and achieve oxidative stress parameters close to the normal range. At a concentration of 5 μmol/l, Kae caused the levels or activities of T-AOC, CAT, and GSH-Px, to increase significantly, whereas the concentration of MDA decreased and the activities of SOD remained unaffected when compared with those of the group treated with H_2_O_2_. The results also demonstrated the increase of Kae on the antioxidant capacity of normal cells (**Figures 2C–G**).

### Kae Ameliorated Cardiovascular Damage and Antioxidant Defense Impairment Induced by Paraquat *In Vivo*

**Figure 3:**
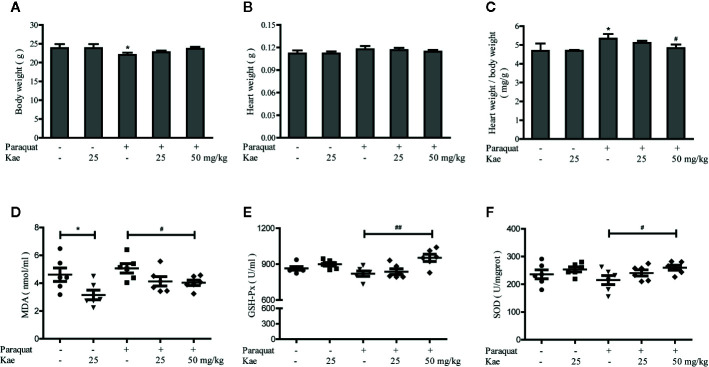
The protective effect of Kae on cardiovascular oxidative stress injury. The effect of Kae on body weight **(A)**, heart weight **(B)**, heart weight/body weight ratio **(C)** treated with or without paraquat. The effect of Kae on the concentration of MDA **(D)** and the activities of GSH-Px **(E)** and SOD **(F)** with or without paraquat. Data are presented as the mean ± standard deviation (SD) of three independent experiments. *P < 0.05, compared with the control group. ^#^P < 0.05 and ^##^P < 0.01, compared with the paraquat-treated group.

Compared with mice in the control group, the body weights of those receiving three injections of paraquat were significantly reduced (P < 0.05). Kae treatment was able to prevent this loss of body weight in a concentration-dependent manner (**Figure 3A**). Although heart weight remained unchanged, the ratio of heart weight to body weight was increased after treatment with paraquat. Kae treatment also inhibited the increase in the ratio of heart weight to body weight compared with mice that were exposed to paraquat alone (**Figures 3B, C**). There was no significant difference in body weight, or in the absolute and relative weight of the heart, between the group receiving 25 mg/kg of Kae and the control group. In our study, we used paraquat to establish a mouse model of cardiovascular oxidative stress; the ability of paraquat to induce oxidative stress has been previously verified in mice. Analysis showed that, compared with that in the control group, the activities of SOD and GSH-Px were reduced in the serum of the model mice, and that the concentration of MDA was increased. Treatment with Kae was able to reduce this oxidative stress in a concentration-dependent manner. Furthermore, 25 mg/kg of Kae was also able to alleviate oxidative stress in normal mice (**Figures 3D–F**). Collectively, these results indicate that Kae can protect blood vessels from oxidative damage induced by chemicals and play a preventive role in maintaining normal cardiovascular structure and function.

### The Effect of Kae on the Levels of Inflammatory Marker Under Normal and Oxidative Stress Conditions *In Vitro* and *In Vivo*

**Figure 4:**
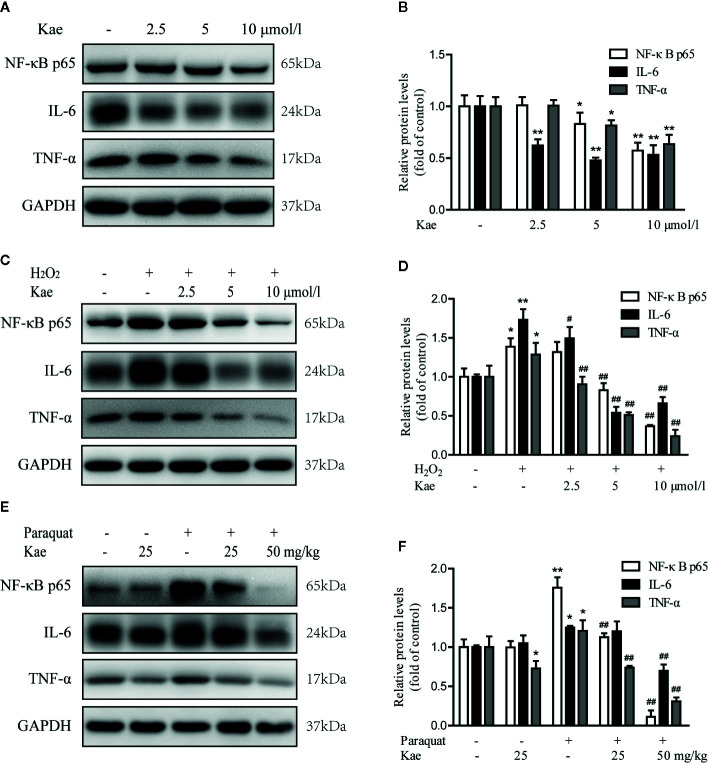
The effect of Kae on the levels of inflammatory markers. **(A, B)** The effect of Kae on the levels of NF-κB p65, IL-6, and TNF-α, in HUVECs. **(C, D)** The effect of Kae and 100 μmol/l H_2_O_2_ on the levels of NF-κB p65, IL-6, and TNF-α in HUVECs. **(E, F)** The effect of Kae and paraquat on the levels of NF-κB p65, IL-6, and TNF-α, in mice. Data are expressed as the mean ± standard deviation (SD) of three independent experiments. *P < 0.05 and **P < 0.01, compared with the control group. ^#^P < 0.05 and ^##^P < 0.01, compared with the model group.

Next, we detected the levels of a series of inflammatory marker (NF-κB p65, TNF-α, and IL-6) by western blotting. Analysis showed that the levels of NF-κB p65, TNF-α, and IL-6, all decreased in a concentration-dependent manner with the application of Kae (**Figures 4A, B**). Furthermore, our results showed that when HUVECs were stimulated with 100 μmol/l of H_2_O_2_, there was a dramatic increase in the expression of NF-κB p65, TNF-α, and IL-6, compared to the levels in untreated cells. However, Kae markedly inhibited the overexpression of these markers (**Figures 4C, D**). Western-blot analysis was used to evaluate the effect of Kae treatment on the levels of these inflammatory markers *in vivo*. Analysis suggested that the levels of NF-κB p65, TNF-α, and IL-6, were significantly upregulated in the abdominal aortas of mice injected with paraquat alone compared to those injected with the vehicle. Kae treatment also significantly attenuated the expression of NF-κB p65, TNF-α, and IL-6, compared with that in mice treated with paraquat alone. However, although Kae reduced the levels of TNF-α, it had no significant effect on the levels of NF-κB p65 and IL-6 in normal mice over the short-term and when administered at a low dose (**Figures 4E, F**).

### The Effect of Kae on the Nrf2/HO-1 Signaling Pathway Under Normal and Oxidative Stress Conditions *In Vitro* and *In Vivo*

**Figure 5:**
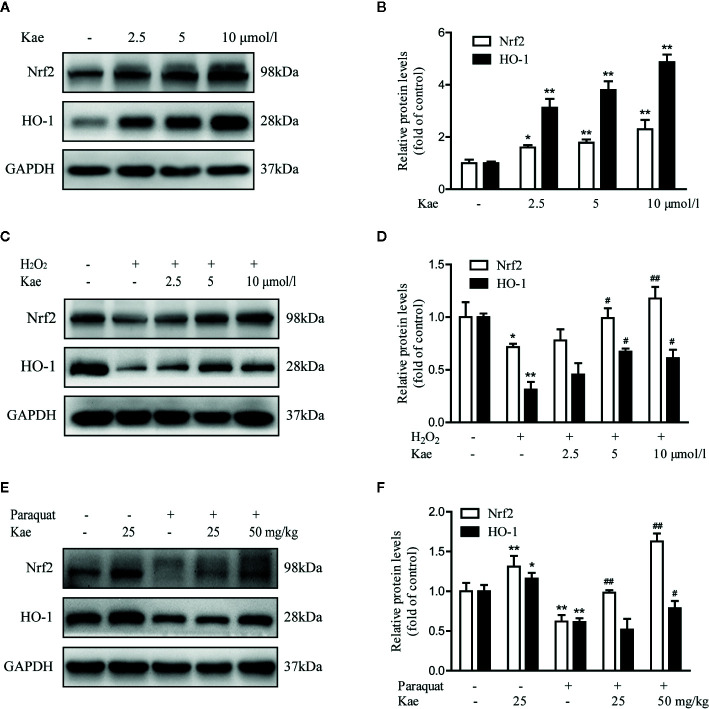
The effect of Kae on the Nrf2/HO-1 signaling pathway. **(A, B)** The effect of Kae on the levels of Nrf2 and HO-1 in HUVECs. **(C, D)** The effect of Kae and 100 μmol/l H_2_O_2_ on the levels of Nrf2 and HO-1 in HUVECs. **(E, F)** The effect of Kae and paraquat on the levels of Nrf2 and HO-1 in mice. Data are expressed as the mean ± standard deviation of three independent experiments. *P < 0.05 and **P < 0.01, compared with the control group. ^#^P < 0.05 and ^##^P < 0.01, compared with the model group.

In order to investigate whether Kae played a role in the activation of the Nrf2/HO-1 pathway, we evaluated the expression of Nrf2 and HO-1 by western-blotting. We found that the Nrf2/HO-1 signaling pathway showed significant levels of activation following Kae treatment (**Figures 5A, B**). Compared with HUVECs that were stimulated with 100 μmol/l of H_2_O_2_ alone, Kae significantly reduced the H2O2-induced down-regulation of the Nrf2/HO-1 signaling pathway (**Figures 5C, D**). Furthermore, we observed that the levels of Nrf2 and HO-1 were significantly elevated in total protein extracts prepared from the abdominal aortas of mice receiving a combination of Kae and paraquat compared with those of mice receiving paraquat alone. However, compared with that in the control group, the expression levels of Nrf2 and HO-1 in the total protein extracts of abdominal aortas from mice receiving Kae alone were only slightly increased (**Figures 5E, F**).

## Discussion

Cardiovascular disease is a serious threat to human life and health. Oxidative stress, endothelial cell activation, and endothelial dysfunction have been widely recognized as major factors in the pathogenesis of vascular diseases (Mittal et al., 2014). Endothelial cells are monolayer cells that are distributed in the vascular lumen and play an important physiological role in vascular homeostasis. Endothelial cell injury is a complex pathophysiological event, involving increased endothelial cell activation and endothelial dysfunction (Liao, 2013). Cytokine-induced endothelial activation under inflammatory conditions often leads to endothelial dysfunction. By regulating the secretion of cytokines, oxidative stress is closely related to endothelial cell activation and endothelial cell dysfunction (Bulua et al., 2011). Kae is an important flavonoid that is commonly found in medicinal plants and plant-derived foods. Kae can also play a key role in antioxidation, anti-inflammation, and anti-apoptosis in a variety of tissues and organs (Wang et al., 2018). However, the specific role of Kae in relieving the inflammation and oxidative stress caused by vascular endothelial injury, and the mechanisms involved, have yet to be elucidated. In view of this, we investigated whether Kae can improve vascular damage by reducing oxidative damage and inflammation. The results of our study strongly indicated that Kae could protect against vascular damage by alleviating oxidative stress and reducing the levels of inflammatory markers. Furthermore, we found that the mechanism involved is related to activation of the Nrf2/HO-1 signaling pathway. In the present study, we used paraquat to induce vascular damage in mice: we found that an intraperitoneal injection of paraquat at a concentration of 10 mg/kg was sufficient to cause inflammation and oxidative stress in vascular endothelial cells and eventually lead to vascular damage.

Most of the current studies on paraquat have focused on lung damage caused by high doses. The increased secretion of proinflammatory factors and proinflammatory mediators in lung tissues that are induced by paraquat have also been reported previously (Amirshahrokhi and Khalili, 2016; Ahmed et al., 2019). Paraquat has also been reported to cause oxidative damage and inflammation in the liver (Amirshahrokhi and Bohlooli, 2013). Recent research suggests that paraquat can induce a phenotype associated with senescence in cardiomyocyte cells by inhibiting the activation of FoxO3, thus leading to cardiac senescence (Chang et al., 2019). In the present study, we demonstrated that paraquat increased the concentration of MDA and reduced the levels of GSH-Px and SOD in the serum of mice. Following treatment with paraquat, mice showed an increase in both the relative and absolute heart weight; these changes were alleviated when paraquat was administered concurrently with Kae. These findings indicate that Kae can alleviate the oxidative stress induced by paraquat in mice, as well as reduce heart weight and the loss in body weight. Consequently, the reduction in paraquat-mediated vascular damage induced by Kae may be related to the up-regulation of antioxidant enzyme activities.

Previous studies have shown that Nrf2 maintains the stability of the intracellular environment by regulating the activity of antioxidant proteins, detoxification enzymes, and other stress response proteins. It has also been established that HO-1 can resist the cytotoxicity of various oxidative stress and inflammatory reactions (Lin et al., 2017; Wu et al., 2017). It is evident that the Nrf2/HO-1 pathway exerts antioxidant and anti-inflammatory effects, reduces mitochondrial damage, regulates cell death and other effects, and ultimately affects the outcome of many diseases. To evaluate the specific involvement of Kae treatment on the levels of Nrf2 in vascular endothelial cells, and its potential role in paraquat-mediated vascular injury, we used immunoblotting to detect the levels of Nrf2 and HO-1. Analysis showed that paraquat caused a significant downregulation of Nrf2 in the abdominal aorta compared with that in vehicle-treated mice. However, the co-administration of paraquat and Kae resulted in a significant increase in the levels of Nrf2 when compared with those in animals treated with only paraquat. These findings are consistent with the role of Kae in reducing oxidative stress *via* the Nrf2/HO-1 pathway.

Inflammation is believed to be an important causative factor for multiple organ damage. The reduction of inflammation by inhibiting the activation of NF-κB has been shown to be an important mechanism with which to mitigate such damage (Yang et al., 2018). Paraquat, the most commonly used non-selective herbicide, is highly toxic to humans and animals (Baltazar et al., 2013), and is known to increase the expression of TNF-α, IL-1β, IL-6, and NO. These factors are common proinflammatory mediators in blood and organs and can often aggravate damage incurred by the body (Donnelly et al., 1994; Ishida et al., 2006). Inhibiting the expression of these proinflammatory mediators has become an important goal for the treatment of paraquat poisoning (Byrne et al., 2016). The p-p65 subunit of NF-kB can be transferred into the nucleus to induce the release of a large number of inflammatory regulators; it can also enhance the inflammatory response and induce organ damage (Amirshahrokhi and Khalili, 2016). In the present study, we confirmed that paraquat induced the over-expression of TNF-α and IL-6, and the activation of NF-κB in the abdominal aorta. However, we also found that Kae alleviated the inflammatory response induced by paraquat. As reported previously, our present data proved that Kae attenuated inflammation and alleviated the progression of vascular injury. Therefore, Kae could alleviate paraquat-induced vascular injury by reducing inflammation *via* the inactivation of NF-κB.

Oxidative stress is known to induce an inflammatory response that aggravates oxidative stress, thus leading to the excessive production of ROS by a range of cells after stimulation (Elmarakby and Sullivan, 2012). Some researchers have speculated that there may be a circulatory relationship between inflammation and oxidative stress (Yang et al., 2018). It has also been reported that endothelial dysfunction caused by the production of ROS often leads to vascular damage (Incalza et al., 2018). The present results confirm that Kae can reduce the levels of ROS and that Kae may help to improve vascular injury by scavenging reactive oxygen species. However, further research is still required to systematically elucidate the specific mechanisms underlying the effects of Kae on the process of vascular injury.

**Figure 6:**
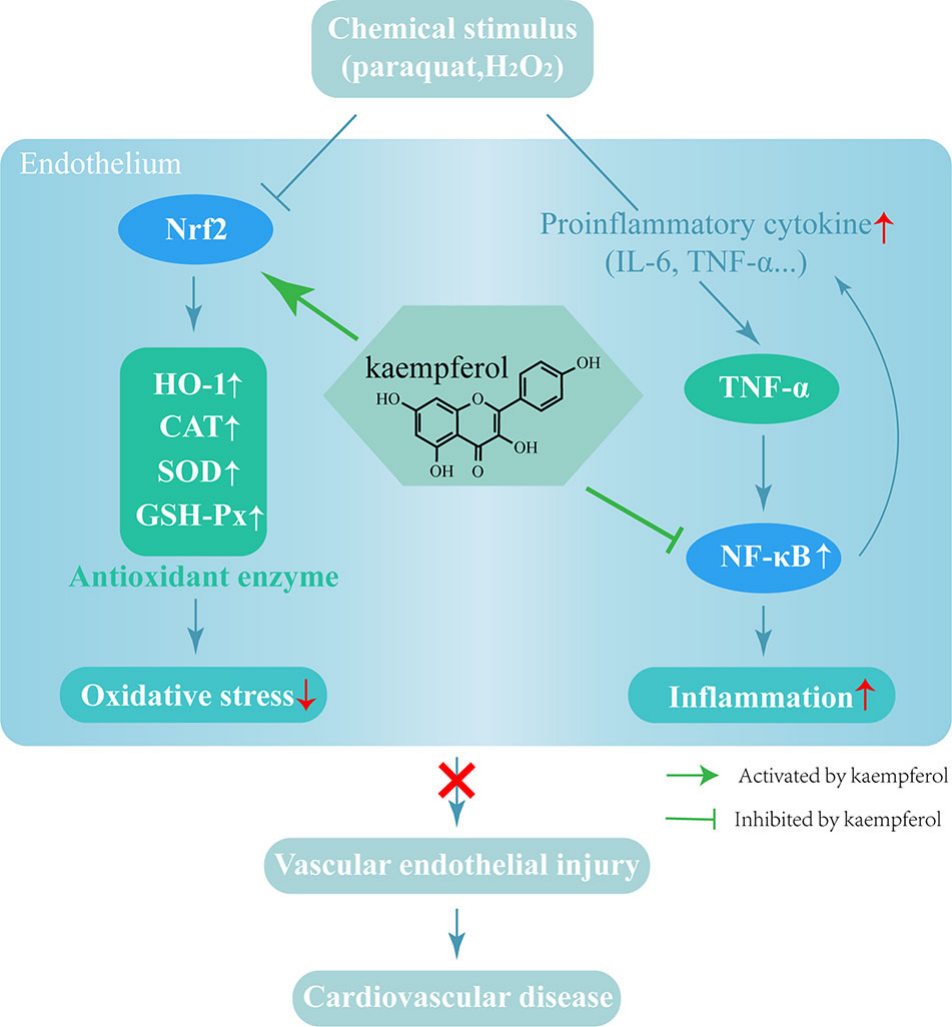
The mechanism by which Kae protects blood vessels from damage.

In summary, the present study used a mouse model to demonstrate that vascular injury led to a significant increase in oxidative damage and the levels of inflammatory factors. Kae was able to alleviate paraquat-induced vascular injury by suppressing inflammation and oxidative stress. Analysis showed that the NF-κB and Nrf2/HO-1 pathways play a key role in these effects. Collectively, our findings demonstrate that Kae is a highly promising candidate for the treatment of acute vascular injury (**Figure 6**).

## Data Availability Statement

The raw data supporting the conclusions of this article will be made available by the authors, without undue reservation, to any qualified researcher.

## Ethics Statement

The animal study was reviewed and approved by the Institutional Animal Care and Use Committee of Beijing Hospital.

## Author Contributions

YL and BC conceived and designed the experiments. HY performed the experiments. JW and XZ were involved in experimental analysis and data acquisition. HY and JS assisted in the western blot analysis and manuscript preparation. YL wrote the manuscript. All authors contributed to the article and approved the submitted version.

## Funding

This work was supported by grants from CAMS Innovation Fund for Medical Sciences (No. 2018-I2M-1-002), the National Natural Science Foundation of China (No. 81671391), and Beijing Hospital Nova Project (No. BJ-2016-033).

## Conflict of Interest

The authors declare that the research was conducted in the absence of any commercial or financial relationships that could be construed as a potential conflict of interest.
